# Multimodal fusion for anticipating human decision performance

**DOI:** 10.1038/s41598-024-63651-2

**Published:** 2024-06-08

**Authors:** Xuan-The Tran, Thomas Do, Nikhil R. Pal, Tzyy-Ping Jung, Chin-Teng Lin

**Affiliations:** 1 GrapheneX-UTS HAI Centre, Australian AI Institute, Faculty of Engineering and Information Technology (FEIT), University of Technology Sydney (UTS), Sydney, NSW 2007 Australia; 2 Electronics and Communication Sciences Unit, Indian Statistical Institute, Calcutta, West Bengal 700108 India; 3 Institute for Neural Computation and Institute of Engineering in Medicine, University of California, San Diego (UCSD), La Jolla, CA 92093 USA

**Keywords:** Human behaviour, Neuroscience

## Abstract

Anticipating human decisions while performing complex tasks remains a formidable challenge. This study proposes a multimodal machine-learning approach that leverages image features and electroencephalography (EEG) data to predict human response correctness in a demanding visual searching task. Notably, we extract a novel set of image features pertaining to object relationships using the Segment Anything Model (SAM), which enhances prediction accuracy compared to traditional features. Additionally, our approach effectively utilizes a combination of EEG signals and image features to streamline the feature set required for the Random Forest Classifier (RFC) while maintaining high accuracy. The findings of this research hold substantial potential for developing advanced fault alert systems, particularly in critical decision-making environments such as the medical and defence sectors.

## Introduction

Anticipating human decision performance is pivotal in predicting potential errors before making a decision, especially in critical fields such as medicine and defence. Psychological studies have developed mathematical models based on signal detection theory^1–3^ and Bayesian decision theory^4^. These models effectively predict decision-making in controlled lab experimental paradigms, such as “go/no go” tasks^5^, ultimatum games^6^, Feelings of Knowing (FOK)^7^, Judgements of Learning (JOL)^8^, Two-alternative Forced Choice tasks (2-AFC)^9^, flanker tasks^10^, higher contrast gabor patches^11^, demand selection tasks^12^, and stroop tasks^13^. The visual searching task is also a widely used paradigm to assess human performance in decision-making^14–17^, where participants aim to locate a target object within an image stimulus as accurately and swiftly as possible. The correctness of the response often serves as a metric for quality decision-making. Beyond behavioural responses, researchers have identified electroencephalography (EEG) as a reliable tool for delving into the neural mechanisms underpinning the decision-making process, courtesy of its high temporal resolution^18^.

The decision-making process primarily involves several key brain regions, many located along the brain’s midline. These regions include the ventromedial prefrontal cortex, anterior cingulate cortex, parietal cortex, frontopolar cortex, and posterior parietal cortex, as highlighted by studies such as Hare et al.^19^, Kolling et al.^20^, Platt and Glimcher^21^, Boorman et al.^22^, Eckstein et al.^14^, and Luck^23^. Specifically, Luck’s research^23^ has been instrumental in detailing the EEG Event-Related Potential (ERP) components and oscillatory activities associated with decision-making in complex visual scenes. This study highlights the critical roles of ERP components such as P1, N1, P2, P3, and N2pc as essential neural markers in processing complex visuals. These components deepen our understanding of the neural mechanisms involved in various decision-making stages and will inform the ERP analysis in this study.

Recent advancements in multimodal approaches aim to refine our understanding of neural mechanisms and improve our ability to decode complex cognitive processes by integrating diverse data types. Several studies have demonstrated promising results in enhancing decoding performance using a multimodal approach that integrates image features with neural data. Palazzo et al.^24^ discovered that a joint brain-image representation significantly improves the performance of Siamese learning networks in tasks such as image classification and saliency detection. Similarly, Du et al.^25^ successfully employed multimodal learning that integrates brain-visual-linguistic features to enhance the neural decoding of visual categories from human fMRI signals. These studies underscore the potential of leveraging multimodal image and neural data features to boost neural decoding capabilities.

Further extending the application of multimodal approaches, research aimed at generating images directly from brain signals also explores the integration of image and neural signals within a latent embedding space. This technique facilitates the correlation between neural activity and image features, thereby enabling the generation of visual representations from brain signals, as explored in works by Chen et al.^26^, Bai et al.^27^, and Sun et al.^28^. However, despite these advancements, the research on combining neural and image features remains relatively limited, particularly for complex tasks such as visual search in intricate contexts.

In addition, while existing experimental paradigms provide significant insights into cognitive activities during human decision-making, they tend to oversimplify the complexity of real-life decision-making scenarios. They typically entail straightforward visual stimuli with two discriminated responses^5,9,14–17^, which may increase the likelihood of guessing correctly by chance (where the probability of the guessed answer being correct is 1/2), potentially undermining the robustness of the analysis outcomes. Consequently, the neural features from such studies may not entirely capture the nuances of neural activity in more complex scenarios. Hence, their applicability in predicting user performance in higher complexity situations remains uncertain. Furthermore, the properties and features of the visual stimuli, such as image complexity and task difficulty, could also support predicting user performance.

Therefore, this study introduces a novel decision-making paradigm based on visual searching. We incorporate EEG brain imaging signals to analyze participants’ decision-making processes in more challenging scenarios. We utilise camouflaged objects to increase the complexity and challenge of the visual search task. This paradigm involves the identification of a camouflaged target object among six sub-regions within an image, reducing the probability of the correct guess response to 1/6. Furthermore, we utilise EEG and image features to predict the correctness of participants’ decision-making. We then apply multimodal methods to combine image (as a source of information) and EEG (as the brain’s response to information) features to predict the accuracy of human decisions in challenging visual searching tasks.

Our study makes several key contributions:

- Introducing a challenging decision-making paradigm that may decrease the likelihood of guessing correctly by offering six options for decision-making.
- Identification of significant EEG features as effective discriminators of decision-making accuracy.
- Proposal of new image feature extraction methods to enhance decision prediction accuracy.
- Demonstration of the superiority of multimodal EEG and image feature fusion over unimodal features in predicting human decision-making performance.

## Results

**Figure 1:**
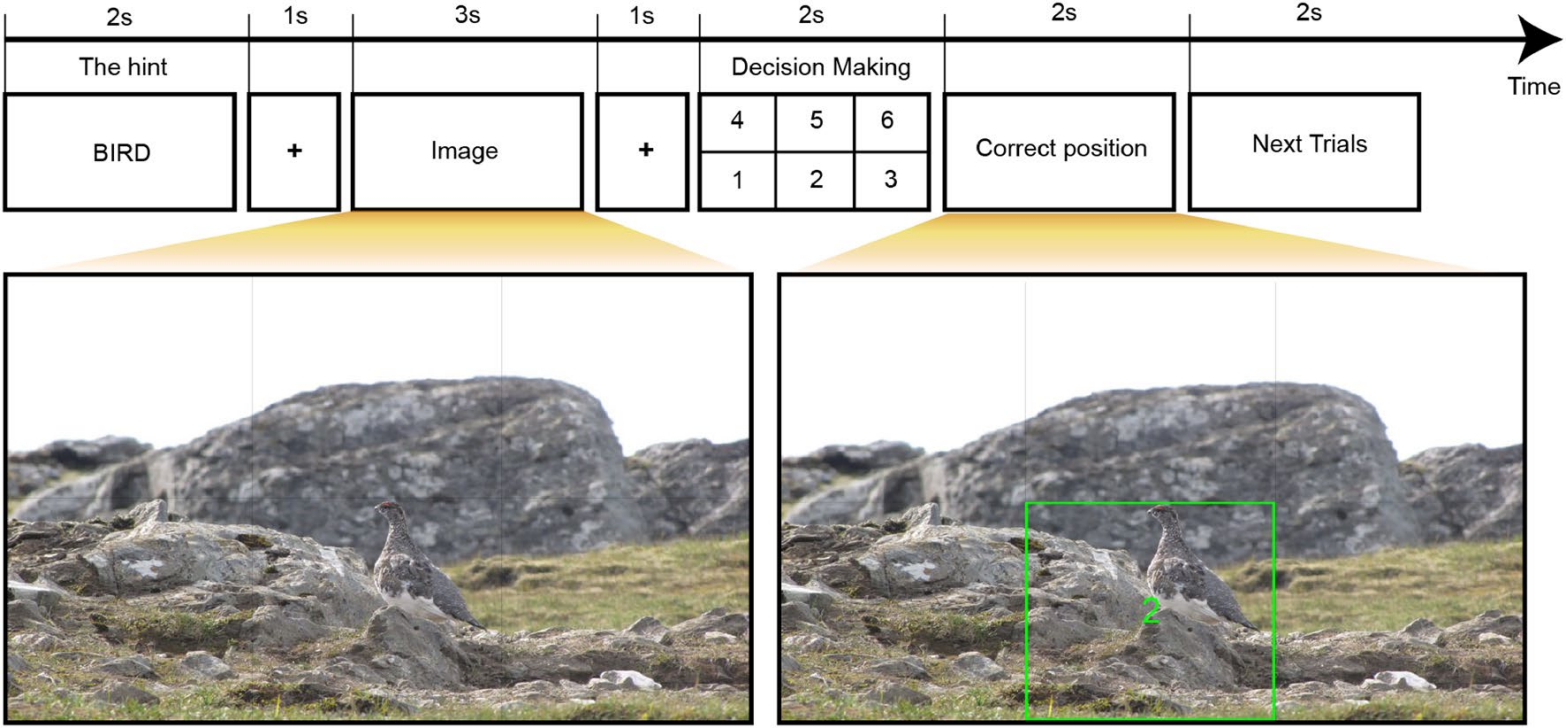
Trial representation: During trials, participants identified an animal in the image, given its species as a hint. Participants indicated the animal’s location by pressing numbers 1–6 on the keyboard. The correct location was then revealed, allowing participants to evaluate their responses. For instance, if the correct location was in region 2, a green bounding box highlighted that region.

**Figure 2:**
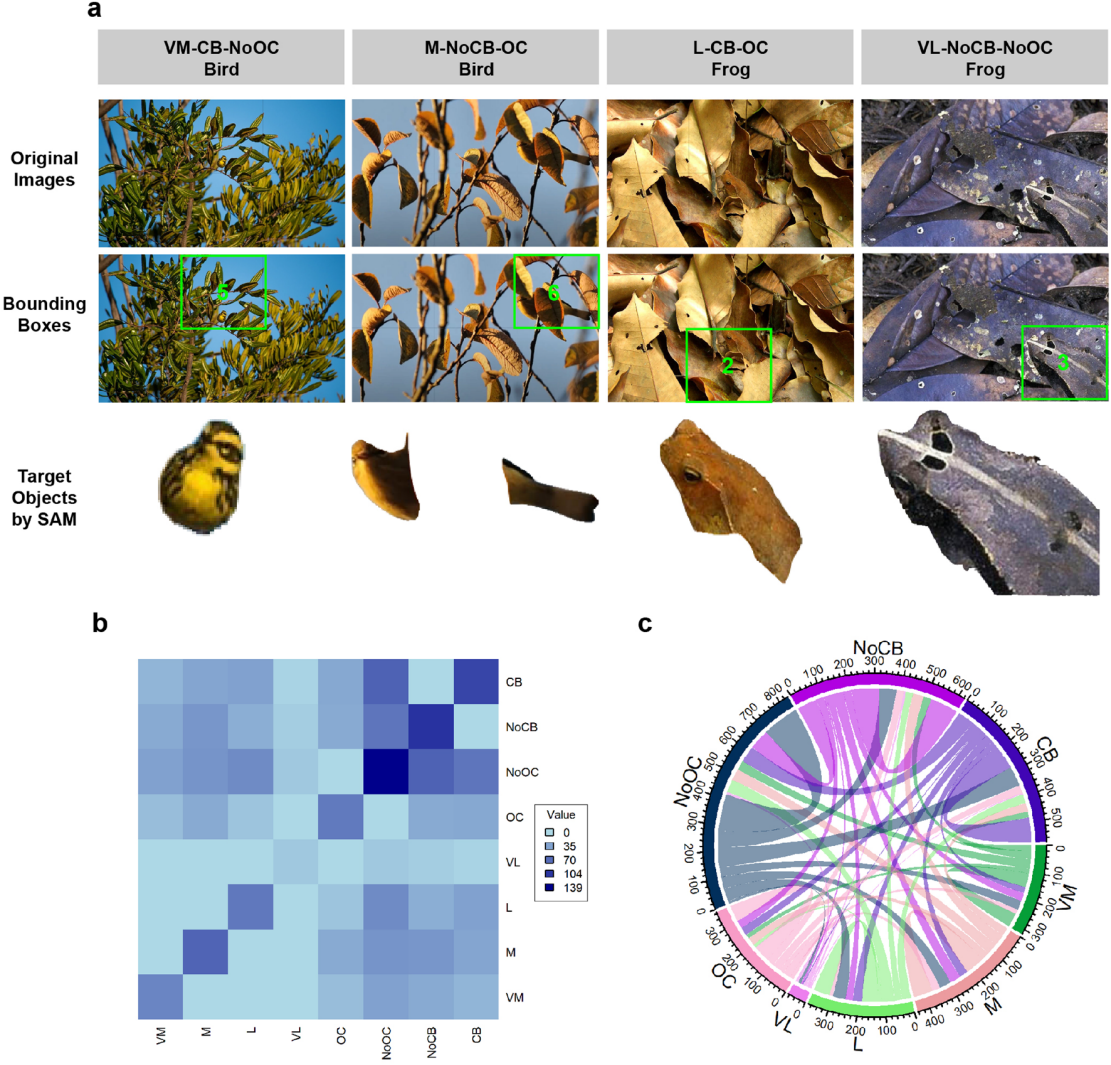
The camouflaged objects in this study share similar colour and shape characteristics with the image background. (**a**) Example images demonstrating various target object characteristics, including target object size (VM—very small, M—small, L—large, and VL–very large), absence of central bias (NoCB—target object positioned in side subregions 1, 3, 4, and 6 of the images), presence of central bias (CB—target object positioned in central subregions 2 and 5 of the images), absence of occlusion (NoOC—target object not covered by another object in the images), and occlusion (OC—target object partially covered by another object). Bounding boxes are used to visually represent the sub-region position of the target object within the images. The example of the target object segmented by the Segment Anything Model (SAM) demonstrates the high quality of the segmentation method, even for small or occluded objects. (**b**) The heatmap displays the distribution of eight target object characteristics (NoCB, CB, NoOC, OC, VM, M, L, VM) within the image dataset. (**c**) The chord diagram depicts the correlation between the eight target object characteristics in the image dataset. The correlation between target object size (L, M, and VM) and other target object characteristics exhibits a well-balanced relationship. In contrast, the correlation involving VL object size is lower possibly due to the limited number of VL target objects in the image dataset. Moreover, while the correlations of CB and NoCB with other target object characteristics are balanced, the correlations involving OC and NoOC tend to be biased towards the NoOC characteristic.

At the beginning of each trial, a 2-s hint revealed the animal’s species, followed by a 1-s fixation period. Then, a 3-s display of the animal image, divided into six grid sections. The participants are required to identify the animal’s location. After a 1-s fixation, participants had 2 s to respond (1–6 on the keyboard). The correct location was highlighted for 2 s, followed by a 2-s rest before the next trial. There were a total of 200 trials in the entire experiment. The image features, and EEG features were extracted during the 3-s display of the animal image to predict participants’ correctness in their responses (see Fig. 1). We selected 200 images from the publicly available camouflaged image dataset COD10K^29^ for image presentation. Figure 2 illustrates some of the images used in our study, showcasing their diverse target object characteristics.

### EEG temporal features

**Figure 3:**
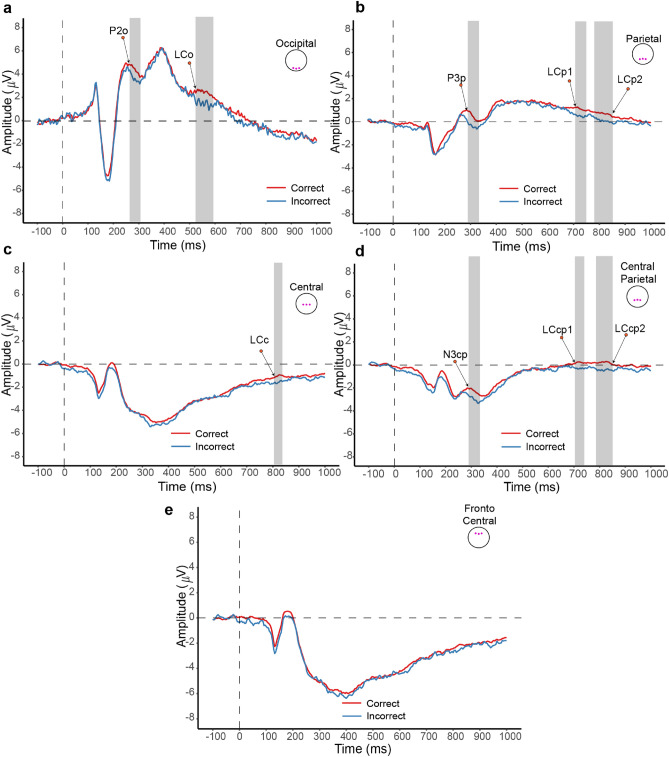
The ERP analysis for correct and incorrect response conditions in 5 brain areas and 12 EEG channels which include the occipital (O1, Oz, O2) channels, the parietal (P1, Oz, P2) channels, the central-parietal (CP1, CPz, CP2) channels, the central (C1, Cz, C2) channels and the frontocentral (FC1, FCz, FC2) channels. The sharding area shows a significant ERP component due to a permutation test coupled with Bonferroni correction (p = 0.05). The ERP component labels include P (Positive), N (Negative), and LC (Late Component). The suffixes o, p, c, and cp denote the brain areas as occipital, parietal, central, and central-parietal, respectively.

We performed grand average event-related potential (ERP) analyses, using a permutation test with Bonferroni correction (in EEGLAB toolbox)^30^, to ascertain which EEG channels and their temporal segments significantly differed between correct and incorrect responses. Figure 3 displays the ’Image Shown’ event ERP components across five brain areas: occipital (O1, Oz, O2), parietal (P1, Oz, P2), central-parietal (CP1, CPz, CP2), central (C1, Cz, C2), and frontocentral (FC1, FCz, FC2) channels. Nine ERP components exhibited statistically significant differences between correct and incorrect responses: P2o and P5o in the occipital area (Fig. 3a), P3p, P7p, and P8p in the parietal area (Fig. 3b), N7c in the central area (Fig. 3c), and N3cp, N7cp, and N8cp in the central-parietal area (Fig. 3d). There is no significant segment difference in the frontocentral region of the ERP (Fig. 3e). Correct response amplitudes were consistently higher than incorrect in all significant ERP components.

These nine ERP components identified in four brain areas (12 EEG channels) led to the extraction of 540 EEG ERP features using five feature extraction methods, as listed in Table 4.

### Identifying key EEG and image features for Random Forest Classifiers

We conducted an important feature analysis to ascertain the most influential EEG and image features for the Random Forest Classifier and select features for multimodal classifier training. Figure 5 presents the top 10 EEG and image features. The most impactful EEG features were from the parietal and central-parietal areas (PZ_P8p_median, CPZ_N8cp_median, CP1_N7cp_median, P2_P8p_rms, PZ_P7p_mean). For image features, SAM features outperformed traditional image features, with the top five beings (Target_object_size_ratio, SAM_segment_density, SAM_the_biggest_segment_dominance, Target_object_occlusion, SAM_number_of_segment). We combined the top five EEG and image features into ten multimodal features for the classifier training.

### Classifier performance using multimodal features versus unimodal EEG and image features

**Figure 4:**
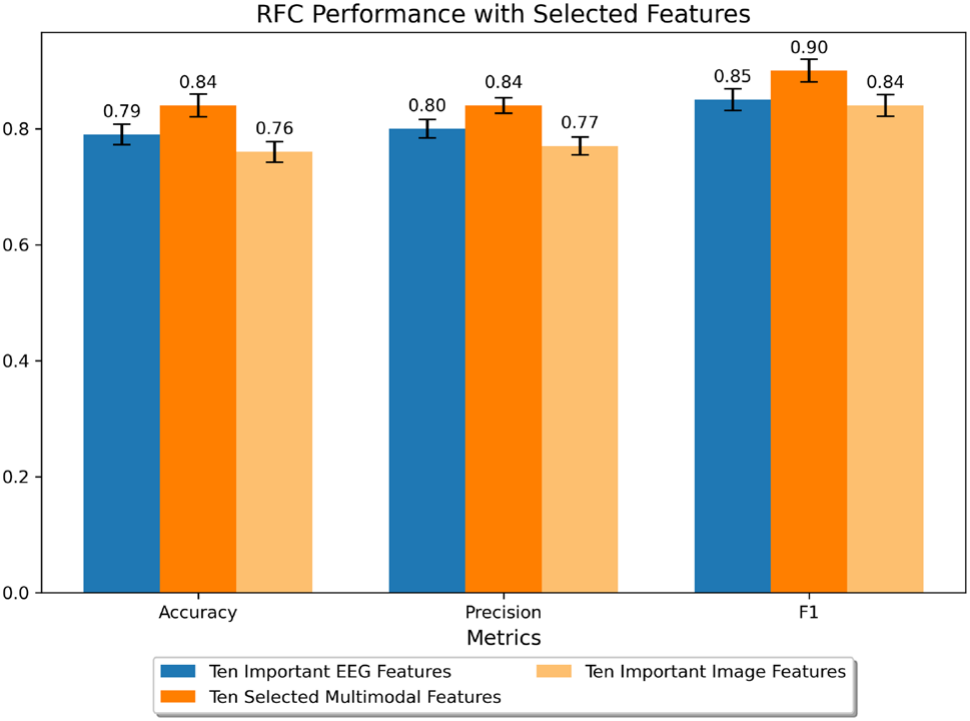
Performance of the Random Forest Classifier trained at the group level with data from 14 subjects, using various feature sets: the top 10 EEG features, the top 10 image features, and a combination of the top 5 EEG and 5 image features as a multimodal approach. Error bars indicate the standard deviation from 5-fold cross-validation.

To analyze the advantages of using multimodal EEG and image features versus each unimodal approach, we trained and tested the Random Forest Classifier at the group level (combining datasets from all 14 subjects for training) using multimodal features and each set of EEG and image unimodal features. We compared the performance of the Random Forest Classifier using the top ten EEG and image features against a set of ten multimodal features, which combines the top five EEG and image features. The results, displayed in Fig. 4, reveal that the selected multimodal features achieve higher accuracy, precision, and F1 scores (0.85, 0.85, and 0.91, respectively) compared to EEG features (0.79, 0.80, 0.85) and image features (0.76, 0.77, 0.84).

### Anticipating human decision accuracy using Random Forest Classifier models

We trained Random Forest Classifiers at the individual subject dataset level using various feature sets to assess the classifier model’s capability to predict participant decisions. Initially, we trained Random Forest Classifiers with all EEG and image features and their combination as multimodal features. We then focused on the top 10 important EEG and image features and a combination of the top 5 EEG and image features. Accuracy was the primary metric for evaluating the model’s alignment with trial labels. We compare the model’s accuracy to the ’reference accuracy,’ which reflects the behavioural performance accuracy of participant decision-making. If the model’s accuracy exceeds the reference accuracy, it indicates that it can effectively identify correct and incorrect participant responses. Table 1 illustrates each subject dataset’s Random Forest Classifier performance. Key findings include:

**Table 1:** Comparative performance of the Random Forest Classifier trained on each subject's dataset (subject level) using all EEG and image features and a multimodal combination of both.

Sub.	Image-features			EEG-features			Multimodal-features			Ref Acc
	Acc	Pre	F1	Acc	Pre	F1	Acc	Pre	F1	
S01	0.85	0.85	0.92	0.88	0.88	0.93	**0.88**	0.88	0.92	0.84
S02	0.93	0.95	0.96	0.93	0.92	0.96	**0.93**	0.93	0.95	0.89
S03	*0.75*	0.74	0.85	0.87	0.88	0.93	**0.85**	0.85	0.90	0.81
S04	0.78	0.79	0.87	0.79	0.79	0.87	**0.80**	0.80	0.88	0.70
S05	*0.80*	0.81	0.87	*0.79*	0.82	0.86	**0.85**	0.85	0.91	0.83
S06	0.81	0.82	0.89	0.83	0.87	0.9	**0.86**	0.88	0.91	0.78
S07	*0.70*	0.70	0.82	0.80	0.80	0.86	**0.80**	0.81	0.86	0.75
S08	0.90	0.90	0.95	0.90	0.90	0.95	**0.92**	0.92	0.96	0.89
S09	0.88	0.87	0.93	0.88	0.89	0.92	**0.89**	0.90	0.93	0.87
S10	0.83	0.86	0.9	0.84	0.85	0.91	**0.86**	0.87	0.91	0.81
S11	0.85	0.87	0.92	0.88	0.88	0.92	**0.90**	0.91	0.93	0.81
S12	0.75	0.75	0.84	*0.70*	0.70	0.82	**0.76**	0.75	0.83	0.71
S13	0.78	0.77	0.87	0.78	0.77	0.87	**0.80**	0.81	0.88	0.76
S14	*0.75*	0.78	0.85	*0.75*	0.75	0.86	**0.78**	0.78	0.87	0.75
Mean	**0.81**	**0.82**	**0.89**	**0.84**	**0.84**	**0.90**	**0.85**	**0.85**	**0.91**	**0.80**

The values in bold represent the highest accuracy, precision, and F1 scores achieved. Cells in italics indicate subject datasets where the classifier did not surpass the reference accuracy (Ref Acc) with the respective feature sets.

1. Multimodal EEG-image features outperformed unimodal EEG (t(14) = 3.05, p = 0.009) and image features (t(14) = 4.52, p = 0.0006) in all subject datasets.
2. The accuracy of Random Forest Classifiers with multimodal EEG-image features surpassed the reference accuracy in all subject datasets (t(14) = 7.34, p = 5.67e–6).
3. EEG features do not show marginally better performance than image features. (t(14) = 1.51, p = 0.15).
4. In comparing unimodal features, the accuracy of the classifiers using image features was not significantly better (t(14) = 1.14, p = 0.28). It failed to surpass reference accuracy in S03, S05, S07, and S14. In contrast, the accuracy of the Random Forest Classifier with EEG features was significantly higher than that of the reference accuracy (t(14) = 3.13, p = 0.008). Random Forest Classifier accuracy with EEG features did not exceed reference accuracy in S05, S12, and S14.
5. The mean Random Forest Classifier accuracy in subject-level training was higher than in group-level training for all image, EEG, and multimodal image-EEG features. Specifically, group-level training using all features resulted in lower accuracy, precision, and F1 scores than single-subject training.

### Efficacy of top important EEG and image features in Random Forest Classifier at subject level

To evaluate whether the top important image and EEG features enhance the performance of the Random Forest Classifier at the subject level and to assess the benefit of their multimodal combination, we trained the classifier on each subject dataset using the top 10 important EEG features, the top 10 important image features, and a set of 10 multimodal EEG-image features. Table 2 details the classifier performance with these selected features. Key observations include:

**Table 2:** Performance of the Random Forest Classifier trained on each individual dataset (subject level) using the top 10 important EEG features, the top 10 important image features, and a set of 10 multimodal features combining both EEG and image data.

Sub.	Image-features			EEG-features			Multimodal-features			Ref Acc
	Acc	Pre	F1	Acc	Pre	F1	Acc	Pre	F1	
S01	*0.84*	0.84	0.90	0.85	0.85	0.89	**0.86**	0.86	0.90	0.84
S02	0.90	0.90	0.91	0.90	0.91	0.92	**0.91**	0.91	0.93	0.89
S03	*0.74*	0.74	0.85	0.82	0.82	0.87	**0.82**	0.82	0.88	0.81
S04	0.77	0.77	0.85	0.75	0.75	0.83	**0.79**	0.80	0.86	0.7
S05	*0.80*	0.80	0.86	*0.76*	0.78	0.84	***0.81***	0.81	0.86	0.83
S06	0.80	0.81	0.87	0.80	0.83	0.88	**0.81**	0.82	0.88	0.78
S07	*0.70*	0.70	0.82	0.76	0.77	0.83	**0.77**	0.78	0.80	0.75
S08	*0.89*	0.90	0.92	*0.88*	0.88	0.91	**0.90**	0.90	0.92	0.89
S09	*0.86*	0.86	0.88	*0.85*	0.85	0.87	**0.87**	0.88	0.90	0.87
S10	0.83	0.85	0.89	0.83	0.84	0.88	**0.83**	0.85	0.90	0.81
S11	0.84	0.86	0.90	0.85	0.86	0.90	**0.85**	0.85	0.90	0.81
S12	0.74	0.75	0.82	*0.70*	0.70	0.82	**0.74**	0.75	0.83	0.71
S13	0.77	0.77	0.82	*0.76*	0.77	0.81	**0.78**	0.80	0.86	0.76
S14	*0.74*	0.75	0.81	*0.73*	0.74	0.82	**0.75**	0.75	0.85	0.75
Mean	**0.80**	**0.81**	**0.86**	**0.81**	**0.81**	**0.86**	**0.83**	**0.84**	**0.88**	**0.80**

The values in bold represent the highest accuracy, precision, and F1 scores achieved. Cells in italics indicate subject datasets where the Random Forest Classifier did not surpass the reference accuracy (Ref Acc) with the respective feature sets.

1. Multimodal EEG-image features demonstrated superior performance over both unimodal image features (t(14) = 2.97, p = 0.01) and unimodal EEG features (t(14) = 4.24, p = 0.009).
2. The Random Forest Classifier's accuracy with multimodal features passes the reference accuracy (t(14) = 3.59, p = 0.003) and passes in 13 out of 14 subject datasets; the exception is the S05 dataset.
3. The Random Forest Classifier’s accuracy with EEG features does not pass that with image features (t(14) = 0.16, p = 0.87).
4. Using unimodal image features, Random Forest Classifier failed to achieve reference accuracy in 7 out of 14 subject datasets, compared to 6 out of 14 when using unimodal EEG features.

### Comparing selected features versus all features in anticipating human decision accuracy

To assess the impact of feature selection on Random Forest Classifier performance, we compared the accuracy of the classifier using all features (as shown in Table 1) versus a subset of selected features (as presented in Table 2). Our analysis focused on the Random Forest Classifier’s performance with the top 10 important features compared to using the complete feature set. We observed a decrease in the classifier accuracy for image features, EEG features, and multimodal features when reducing the number of features, as confirmed by t-test results.

## Discussion

**Figure 5:**
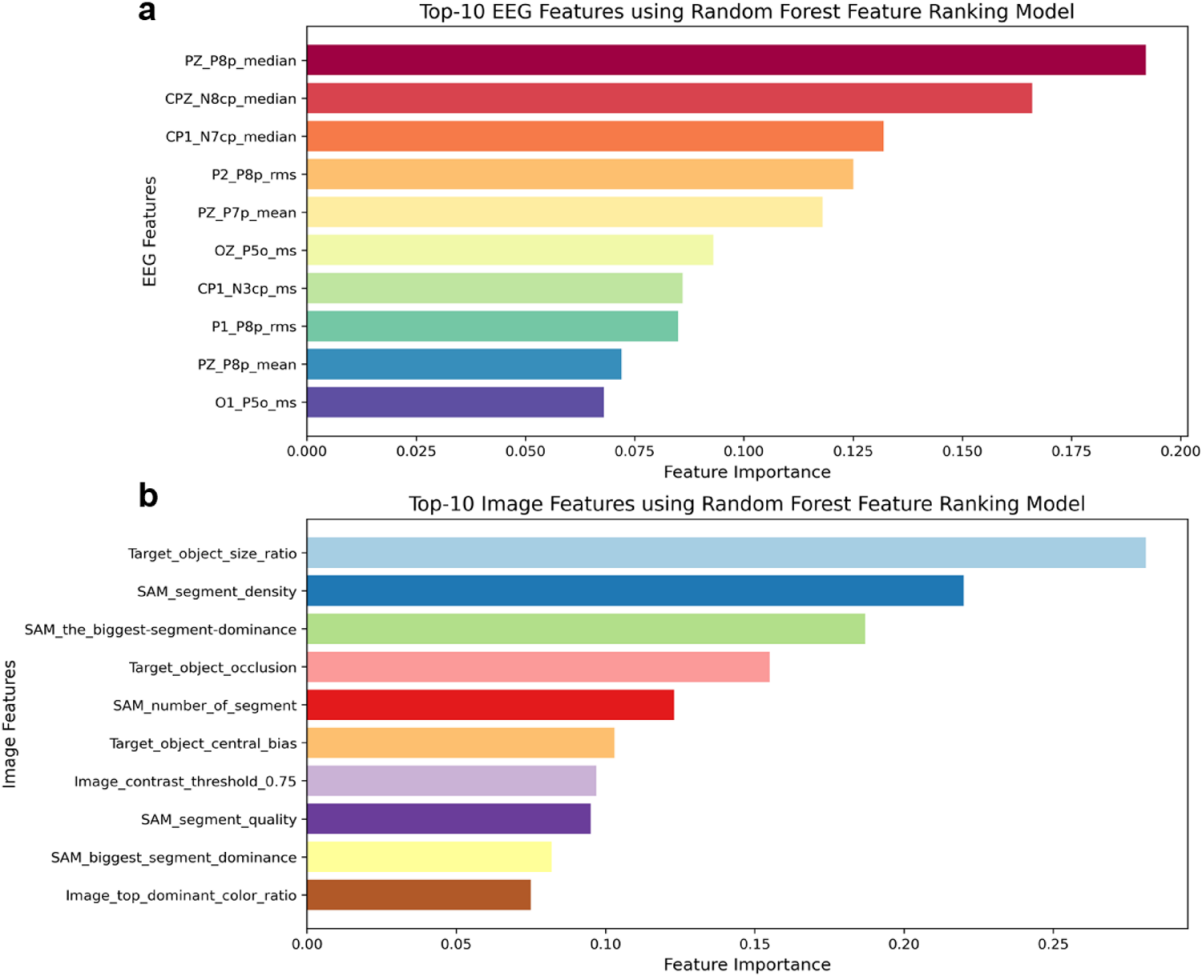
Top 10 key image and EEG features identified through Random Forest Classifier important feature analysis.

This study’s primary objective was to identify significant EEG features capable of distinguishing between correct and incorrect decisions. The ERP analysis highlighted key segments within the occipital, parietal, and central-parietal brain areas as crucial discriminators for predicting decision accuracy (as shown in Fig. 3). Further analysis to identify important EEG features for Random Forest Classifiers emphasized the significance of EEG channels in the parietal area (as shown in Fig. 5). This finding aligns with prior research underscoring the pivotal role of the parietal cortex in visual search and decision-making tasks^14,23,31–33^.

The second significant aim of this study was to assess the predictive power of image features on decision-making outcomes. By employing traditional and innovative feature extraction methods, including the SAM, we examined the influence of image-based information on decision correctness. The SAM method, which clarifies the relationship between basic image and target object features, was notably effective for the classifier. This highlights the predictive value of visual information in cognitive outcomes, resonating with the findings of Li et al.^34^, and Iigaya et al.^35,36^, who demonstrated the potential of image features in classifying visual quality and predicting participant choice behaviors, respectively. Our approach extends these methodologies, illustrating that detailed image analysis can provide significant insights into decision-making processes.

The third objective was to determine if a multimodal fusion of EEG and image features could outperform the predictive capability of unimodal features in forecasting decision-making accuracy. By rigorously training the classifier with both multimodal and unimodal feature sets, we consistently observed the superiority of the multimodal approach. This finding aligns with the growing consensus in cognitive neuroscience and machine learning^37^ that integrating multiple data sources can substantially improve model accuracy. Similar benefits have been reported in studies utilising multimodal data, such as EEG and eye-tracking for emotion and attention classification^38,39^, and EEG with facial expressions or speech signals for emotion recognition^40–43^. Our results further affirm the effectiveness of multimodal feature integration, indicating a promising direction for enhancing predictive models in cognitive science and decision-making research.

We introduced a novel experimental paradigm for decision-making centred around visual search tasks. This paradigm, designed to address the limitations of traditional discriminatory decision-making tasks, notably the high likelihood of correct guesses, by incorporating camouflaged objects as the target. This approach increases the complexity of the tasks and demands greater attention from participants, thereby eliciting more distinct cognitive patterns essential for our analysis. Such methodological innovation is key in creating a task environment that challenges participants and evokes robust neurophysiological markers of decision-making processes.

By engaging in a demanding visual search task with camouflaged targets, we venture into an area that might not fully align with existing findings on brain dynamics in decision-making. Our methodology bears similarities to Luck’s study^23^, which also explores the decision-making process within complex visual scenes. This research emphasises the significant role of EEG components, primarily observed in the posterior brain regions, in visual processing. Our findings affirm the involvement of these components in the visual search tasks our subjects undertook, and we also identified late positive potential (LPP) and late negative potential (LNP) components, which we believe are related to the decision-making process and the subjects’ reassessment of their decisions. This interpretation is supported by recent studies^44^, enriching our comprehension of the neural underpinnings of decision-making in visually complex tasks.

Furthermore, this study compares the classifier’s performance when using unimodal EEG features versus image features and between group-level and subject-level training. Our results demonstrate that EEG features consistently outperform image features, a trend we attribute to the dynamic nature of EEG data. Unlike static image features, which lack temporal information, EEG data is continuously collected throughout trials, capturing the brain’s rapid responses and the variability inherent in the trials and among participants. This rich temporal information provides a more detailed understanding of brain activity related to decision-making accuracy. The distinction in performance between subject-level and group-level training is driven by the variability across participants and experimental conditions. While this variability poses challenges in generalising findings across a group, it offers valuable insights when analysed at the individual level, potentially leading to more personalised approaches and a deeper understanding of individual cognitive processes.

Subject-level analysis across participant datasets validates the effectiveness of employing EEG features, image features, and their multimodal fusion to predict decision accuracy. The application of a multimodal approach, integrating a comprehensive set of EEG and image features, resulted in the classifier accuracy with multimodal EEG-image features surpassing reference accuracy across all subject datasets (t(14) = 7.34, p = 5.67e−6). Additionally, when the multimodal fusion was limited to the top 5 EEG and top 5 image features, RFC accuracy with multimodal features continued to exceed reference accuracy (t(14) = 3.59, p = 0.003) in 13 out of 14 subjects' datasets except for subject S05 (as shown in Fig. 2). These findings highlight potential applications for fault alert systems that could proactively indicate the probability of erroneous decisions, especially during critical decision-making stages. Such systems could be invaluable in high-risk sectors such as healthcare and defence, where the consequences of mistakes are particularly severe.

Nonetheless, this study has several limitations that warrant attention. First, the relatively modest dataset, comprising only 14 subjects, might limit how much our ERP findings can be generalised and could affect the stability or reliability of classifier training outcomes at a group level. Second, while image features provide insights into the challenges of detecting target objects, a more comprehensive behavioural experiment with varied difficulty levels and controlled conditions is necessary to determine the complexity of each image accurately. Such data would enable a more nuanced analysis of brain dynamics in relation to object detection tasks. Looking ahead, we plan to expand and diversify our EEG and behavioural data collection to address these limitations and enhance the robustness of our findings.

In summary, our research effectively demonstrates the utility of leveraging multimodal EEG and image features to predict the accuracy of human decisions. Our results show that EEG features, particularly from the parietal cortex, significantly enhance the discriminative capability of classification models, as evidenced by improved classification metrics. Furthermore, we introduced the SAM as a technique for extracting image features, which has proven beneficial in enhancing classifier performance. By incorporating camouflaged objects to simulate real-world complexity in visual search and decision-making tasks, our experimental paradigm closely mirrors the challenges encountered in actual decision-making situations, necessitating increased participant engagement. These insights pave the way for developing sophisticated fault alert systems designed to preemptively signal potential human errors based on predictive models of decision-making accuracy.

## Methods

### Participants

Fourteen healthy subjects, including one female, participated in this study (aged 20–38 years, mean ± SD: $25.1 \pm 4.2$; 2 left-handed), all of whom had normal or corrected-to-normal vision. All participants provided informed consent, which was reviewed and approved by the ethical committee of the University of Technology Sydney, Australia (approval Grant number: UTS HREC REF NO. ETH22-7038). The study was conducted in accordance with the relevant guidelines and regulations.

### Image dataset and experimental paradigm

Two hundred images were selected from the publicly available camouflaged image dataset COD10K^29^. These images feature a single animal in a challenging scene for detection. To enhance visual clarity and minimize head movements while searching for animal object, all images were resized to dimensions of $1000 \times 600$ pixels. Figure 1a illustrates the process of each trial. At the beginning of each trial, a hint displaying the animal’s species was shown for 2 s, followed by a 1-s fixation period. Subsequently, the image containing the animal was displayed for 3 s, partitioned into six equal-sized areas by thin grid lines. Participants were instructed to locate and indicate the region where the animal was situated. After a 1-s fixation period, they had 2 s to respond with their decision by pressing a number from 1 to 6 on the keyboard. Once they made their choice, the correct location of the object was highlighted for 2 s, followed by a resting period of 2 s before the commencement of the next trial.

In total, there were 200 images in this experiment. The experiment was divided into four blocks, with 50 trials in each block. There were no repeated images throughout the entire experiment. Each block took 10 min, with a 5-min break between blocks. Before the experiments, the participants completed a practice test comprising ten trials to familiarize themselves with the task. The experiment lasted approximately 1 h.

### EEG data acquisition

The EEG data were recorded with a Neuroscan Synamps 2 amplifier and 64-channel Quik-Cap (Compumedics, Australia). The impedance in all channels was maintained below $5k\Omega$. The EEG data were sampled at a rate of 1000 Hz. An HP 27-in. (resolution $1920 \times 1080$ and fresh rate 60 Hz) screen was utilised, with the distance between the screen and the participant’s headset at 40 cm. The images were displayed at the centre of the screen to minimise head and body movement during the experiment when searching for the target object.

### Experiment application

The experiment was conducted using Unity Version 2020.3.29f1. The application streamed an event marker via UDP networking and recorded button presses using the Unity event library, detailing the event onset firing time and the button pressed. A separate CSV file containing metadata about the images used in each trial was utilised by Unity to load the images. This included the image ID, trial number, and the precise location of the target object. We compared this metadata with the participant’s performance file to categorise trials as correct or incorrect. If the participant accurately identified the location, the term ‘Correct’ was entered into a specific column in the participant’s performance file. Upon completing all 200 trials, the image metadata file and the participant’s performance file were merged to form a comprehensive behaviour dataset for each participant. The ’Correct’ column in this dataset was then used as a label to train the models on the images and EEG features.

### EEG data preprocessing

We used the EEGLAB toolbox v14.1.2^30^ to pre-process the recorded EEG data, following the methodology adapted from^45^. Initially, the EEG data were downsampled to 250 Hz, processed through a high-pass filter at 1 Hz and subjected to line-noise removal. Post-filtering, the data were average-referenced and subjected to adaptive mixed independent component analysis (AMICA)^46^. Eye components were identified using the ICLabels toolbox^47^ and removed.

Following this, epoch data were extracted, commencing from the onset of the image presentation and spanning a duration of [$-100$, 1000] milliseconds (ms). These epochs were categorised into two groups based on the participants’ responses: correct and incorrect. The epochs within each group were then averaged. The epoch extraction process were conducted using the Python MNE toolbox v1.3.0^48^.

### EEG ERP segment selection

To identify significant EEG features that could effectively discriminate between correct and incorrect response classifications. We conducted grand average ERP analyses and applied a permutation test with Bonferroni correction^30^. Selecting significant ERP segments identifies those segments displaying marked differences in ERP values between correct and incorrect response conditions. The permutation test, a non-parametric approach, was chosen for its advantage of not requiring the assumption of a normal distribution in the data. To determine the p-value, we calculated the proportion of permutations in which the observed differences were as extreme as or more extreme than those in the baseline period. This method thoroughly evaluates the statistical significance of the differences between correct and incorrect responses.

Given the multiple comparisons inherent in analyzing multiple time points, we applied the Bonferroni correction to control the family-wise error rate. This correction adjusted our significance threshold by dividing it by the number of comparisons. Specifically, if ’p’ represents the original significance level (we used p-value = 0.05), and ’m’ denotes the number of time points tested, the adjusted significance level was set at p/m. This rigorous approach mitigated the risk of type I errors (false positives) due to multiple testing. However, it also increased the likelihood of type II errors (false negatives), a common trade-off in statistical correction methods. As a result, Table 3 presents the significant EEG ERP segments identified from channels across various brain areas. From Table 3, one may say that the features Mean Square and Standard Deviation are very similar and only one could be enough. However, while designing a classifier, one of the features may be more helpful to find the classification boundary than the other. Hence, we have kept both.

**Table 3:** Selection of significant EEG ERP segments from channels across different brain areas.

ERP components	Brain-area/channels	Segment (ms)
P2o	Occiptial—(O1, Oz, O2)	51 (265–306)
LCo	Occiptial—(O1, Oz, O2)	74 (521–595)
P3p	Parietal—(P1, Oz, P2)	48 (295–343)
LCp1	Parietal—(P1, Oz, P2)	45 (705–750)
LCp2	Parietal—(P1, Oz, P2)	82 (785–867)
LCc	Central—(C1, Cz, C2)	33 (802–835)
N3cp	Central-Parietal—(CP1, CPz, CP2)	50 (295–345)
LCcp1	Central-Parietal—(CP1, CPz, CP2)	34 (702–736)
LCcp2	Central-Parietal—(CP1, CPz, CP2)	56 (794–850)

The values in the ’Segment length’ column represent the duration of each segment, with specific duration information provided in parentheses.

### EEG feature extraction

The significant segments delineating the differences between conditions in the prior step were subsequently utilised for EEG feature extraction. We pinpointed 9 significant segments, as illustrated in Fig. 3. We employed five distinct temporal feature extraction techniques for each segment as detailed in Table 4. These techniques were applied to channels in five principal brain regions: the occipital (O1, Oz, O2) channels, the parietal (P1, Oz, P2) channels, the central-parietal (CP1, CPz, CP2) channels, the central (C1, Cz, C2) channels, and the fronto-central (FC1, FCz, FC2) channels. The selection of the five brain regions for ERP segment analysis was informed by a comprehensive review of related literature, which identified these areas as significant in cognitive processes related to decision-making. Choosing three distinct channels within each region was a strategic decision to prevent channel overlap across regions, ensuring that the data reflects region-specific activity. This approach also helps to manage the volume of EEG features, avoiding an excessive number that could complicate the analysis and model training. This strategy resulted in a compilation of 540 EEG features, computed as 9 segments $\times$ 12 channels $\times$ 5 methods. These features were prepared for input into the Random Forest Classifier model. To ensure feature independence within the training set, we conducted a correlation analysis on the EEG features, allowing for the removal of any interdependent features. We established a correlation threshold of 0.80 to eliminate dependent features, resulting in the selection of 481 EEG features for training the RFC model.

**Table 4:** EEG features, definition, and their mathematic equations.

Features	Equations	Definition
Mean	$\bar{x} = \frac{\sum _{i=1}^nx_i}{n}$	The sum of the amplitudes of all data points $x_i$, divided by the total number of data points (n) in each epoch
Median	$m=x_{(n/2)}$	The amplitude value of the middle position $x_{(n/2)}$ in sorted set of n data points in each epoch
Standard deviation	$s = \sqrt{\frac{1}{n-1} \sum _{i=1}^n (x_i - \overline{x})^2}$	The dispersed of data points’ amplitude value $x_i$ to the mean amplitude value of n data points in each epoch
Mean square	$ms = \frac{1}{n} \sum _{i=1}^{n} (x_i - \bar{x})^2$	Sum of all time point’s amplitude values $x_i$ divided by the total number of time points (n) in each epoch
Root mean square	$rms = \sqrt{\frac{1}{n} \sum _{i=1}^{n} x_i^2}$	Sum of the squared amplitude of all data points $x_i$ divided by the number of data points (n) in each epoch

Each participant engaged in 200 trials, creating a training set labelled a column vector comprising 200 binary (0 or 1) entries. In this context, a zero denotes an incorrect trial, while a one indicates a correct trial.

### Image feature extraction

In our research, we have employed both conventional image feature extraction techniques, focusing on the inherent characteristics of images and the properties of target objects, and we have innovated a novel method for image feature extraction using the SAM. This dual approach allows us to analyze images from a traditional perspective while leveraging cutting-edge technology to extract more complex and potentially informative features related to object relationships within the images. The SAM-based method is particularly adept at identifying and segmenting specific features within an image, which can be crucial for detailed visual analysis tasks.

#### Basic image features

Seven fundamental image features were extracted from every pixel for each image in the sample. These include the mean hue, saturation, and brightness values, which provide a basic colour profile of the image. Additionally, the proportions of the top dominant and the top three dominant colours were calculated to capture the most prevalent colour patterns. Two contrast thresholds were applied, with ratios established at threshold values of 0.75 and 0.85, to measure the contrast intensity. Finally, an assessment of the overall image quality was included to gauge the clarity and detail in the images.

1. HSV mean features: Mean-hue, mean-saturation, and mean-brightness were captured from the Hue, Saturation, and Brightness (HSV) values^49^. Hue refers to the dominant colour family, which was based on the primary colours of the RGB model. Saturation describes the intensity of the colour. For example, a grey-scale or black-and-white photo has no colour saturation, while a full-colour photo of a field of bright wildflowers might be highly saturated. Brightness is the relative lightness or darkness of a particular colour, from black (no brightness) to white (full brightness). The base HSV values for all pixels were calculated by the following equation from^49^:
  1 $$\begin{aligned} H = arccos\frac{\frac{1}{2}(2R-G-B)}{\sqrt{(R-G)^2 - (R-B)(R-G)}}\end{aligned}$$
  2 $$\begin{aligned} S = \frac{max(R,G,B) - min(R,G,B)}{max(R,G,B)} \end{aligned}$$
  3 $$\begin{aligned} V = max(R,G,B) \end{aligned}$$
  Then, the mean-hue, mean-saturation, and mean-brightness values were calculated as the means of the HSV values across all pixels in the image:
  4 $$\begin{aligned} H_{mean} = \frac{1}{MN}\sum _{n}\sum _{m}H(m,n) \end{aligned}$$
  5 $$\begin{aligned} S_{mean} = \frac{1}{MN}\sum _{n}\sum _{m}S(m,n) \end{aligned}$$
  6 $$\begin{aligned} B_{mean} = \frac{1}{MN}\sum _{n}\sum _{m}V(m,n) \end{aligned}$$
  where *M* and *N* are the number of rows and columns in the image, and *H*(*m*, *n*), *S*(*m*, *n*), *V*(*m*, *n*) are, respectively, the hue, saturation, and brightness values at pixel (m,n) of the image.
2. Colour dominant features: Our hue value refers to the image’s dominant primary colour family, but the COD10K dataset images are usually not very diverse in colour. In any image, the background colours and objects within the picture are often very similar. Thus, we defined the next two basic image features of the dominant colour distribution. Here, we turned to the K-means method in the Sklearn Cluster library and clustered the colours’ RGB values into ten groups for each image^50^. The output result is the percentage of each colour occupied in the image. From this, we calculated two ratios as features: the most dominant colour and the top 3 most dominant colours, as defined in Eqs. (7) and (8). The most dominant colour is usually the background, while the top 3 most dominant colours often include the background and some colours in the target object.
  7 $$\begin{aligned} f_{\text {top-dominant-color-ratio}} = \text {max(Kmean color cluster's ratio)} \end{aligned}$$
  8 $$\begin{aligned} f_{\text {top-3-dominant-color-ratio}} = \text {sum(top three Kmean color clusters' ratio)} \end{aligned}$$
3. Contrast features: Another basic image feature we extracted was contrast. Contrast is determined by the distribution of colour in the image. The histogram for high-contrast images usually spans a broader value range, while the histograms for low-contrast images cover a narrow range. We used the “is low contrast” function from the scikit-image libraries^51^ with two threshold values, 0.75 and 0.85, as depicted in Eqs. (9) and (10), respectively. These threshold values helped to classify the images into low and high-contrast categories. We tried many thresholds and decided on 0.75 and 0.85 because they resulted in a good relative ratio of low-contrast images (30% and 50%).
  9 $$\begin{aligned} f_{\text {contrast-threshold-0.75}} = {\left\{ \begin{array}{ll} 0 &{}\text {if contrast value} < 0.75. \\ 1 &{}\text {otherwise}. \end{array}\right. }\end{aligned}$$
  10 $$\begin{aligned} f_{\text {contrast-threshold-0.85}} = {\left\{ \begin{array}{ll} 0 &{}\text {if contrast value} < 0.85. \\ 1 &{}\text {otherwise}. \end{array}\right. } \end{aligned}$$
4. Image quality feature: The seventh and last basic image feature is image quality. To gauge this value, we used the blind/referenceless image spatial quality evaluator (BRISQUE)^52^ method. BRISQUE is a model that only uses image pixels to calculate features. Other methods are based on transforming the image into other spaces, like wavelet or DCT; hence, the BRISQUE method is much more efficient computationally. The BRISQUE output for each image is from 0 to 100, with a lower value indicating a higher image quality.
  11 $$\begin{aligned} f_{\text {image-quality}} = \text {n for n in (0, 100).} \end{aligned}$$

#### Target object features

Camouflaged objects are the focal points in visual search tasks, making features derived from these target objects crucial due to their representation of central stimulus properties. We focus on three key features of target objects: object size ratio, object occlusion, and object central bias.

1. Object size ratio feature: Some features pertaining to the target object are included in the metadata of the COD10K dataset. The target object size is graded into four levels—very small (0), small (1), large (2), and very large (3)—based on the ratio between the object and the image as computed in Eq. (12).
  12 $$\begin{aligned} f_{\text {object-size-ratio}} = {\left\{ \begin{array}{ll} 0 &{}\text {if object-size-ratio}< 1/48. \\ 1 &{}\text {if} 1/48 \leqslant \text {object-size-ratio}< 1/24. \\ 2 &{}\text {if} 1/24 \leqslant \text {object-size-ratio} < 1/12. \\ 3 &{}\text {if} 1/12 \leqslant \text {object-size-ratio} \leqslant 1/6. \end{array}\right. } \end{aligned}$$
2. Object occlusion feature: Object occlusion refers to whether the target object is partially covered by another object in the image. This is a binary feature as in Eq. (13):
  13 $$\begin{aligned} f_{\text {object-occlusion}} = {\left\{ \begin{array}{ll} 0 &{}\text {if no occlusion.} \\ 1 &{}\text {otherwise}. \end{array}\right. } \end{aligned}$$
3. Object central bias feature: Central bias concerns a human’s instinct to focus on the middle of the image to look for something^53^. We divided the images into six equal regions, with regions 2 and 5 occupying the top and bottom centre of the image, respectively. Regions 1, 3, 4, and 6 are on the sides. Again, this was recorded as a binary variable as in Eq. (14):
  14 $$\begin{aligned} f_{\text {object-central-bias}} = {\left\{ \begin{array}{ll} 0 &{}\text {if object lies in sub-regions 1, 3, 4 and 6}. \\ 1 &{}\text {if object lies in sub-regions 2 and 5}. \\ \end{array}\right. } \end{aligned}$$

#### SAM relationship features

Li and Chen^34^ employed the Graph Cut segmentation method to segment objects in paintings. However, in images containing concealed objects, like those in the COD10K dataset^29^, there are numerous potential segments (objects), rendering traditional segmentation methods less effective. Consequently, we adopted the SAM^54^, developed by the MetaLab research team.

**Figure 6:**
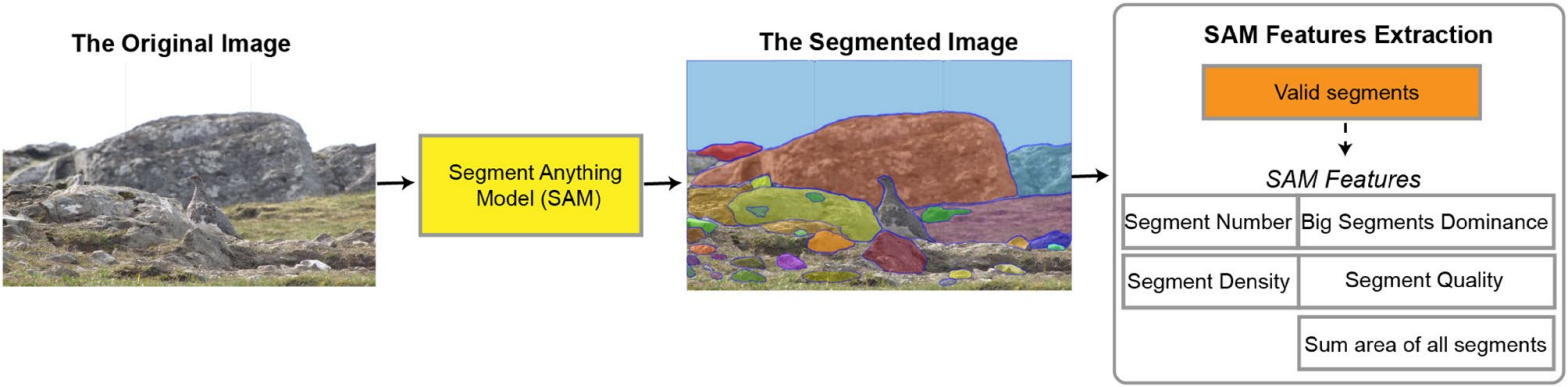
SAM feature extraction pipeline. SAM model is fine-turned for our image dataset to extract the SAM image features. The SAM model segments all the objects in the image into valid segments. From the segment data, four SAM features are extracted: the number of segments (objects) in the image, segment dominance (the largest objects), segment density, and segment quality.

Utilising outcomes from the SAM, we computed seven relational features to assess the interaction between each image and its contained objects. These features encompass the count of segments, the cumulative area of all segments, the dominance of the largest segment, the density of segments, and the quality of segmentation, offering insights into the spatial arrangement and clarity of objects within the images. The process of extracting SAM (Segment Anything Model) features is depicted in Fig. 6.

1. Number of segments feature: The initial feature is the number of segments in the image, corresponding to the count of masks outputted by SAM.
  15 $$\begin{aligned} f_{\text {number-of-segment}} = \text {the number of SAM output masks} \end{aligned}$$
2. Sum of segment area feature: The feature corresponds to the sum area of all valid segments outputted by SAM.
  16 $$\begin{aligned} f_{\text {sum-of-segment-area}} = \text {sum(area of all SAM output segments)} \end{aligned}$$
3. The biggest segment dominance feature: The feature corresponds to the ratio between the area of the biggest segment to the overall segment area computed in Eq. (16).
  17 $$\begin{aligned} f_{\text {the-biggest-segments-dominance}} = \frac{\text {the biggest segment area}}{\text {sum(segments area)}} \end{aligned}$$
  It’s important to note that in our dataset, all images are uniformly sized, allowing the segment size (or the pixel count in a segment) to be a sufficient metric for identifying dominant segments. These larger segments tend to draw more attention during the search process and are significantly linked to the size feature within the target object feature set.
4. Segment density: It postulates that higher segment density in images complicates the task of locating the target object. This metric is calculated by dividing the total Euclidean distance between centroids of all pairs of SAM segments by the total number of SAM segments, as shown in Eq. (18).
  18 $$\begin{aligned} f_{\text {segment-density}} = \frac{\text {sum(distance of SAM segment pairs)}}{\text {number of SAM segments}} \end{aligned}$$
5. Segment quality feature: The final feature in this set is the mean segment quality as provided by SAM, determined by dividing the sum of the quality scores of all segments by the number of segments:
  19 $$\begin{aligned} f_{\text {segment-quality}} = \frac{\text {sum(segment's quality value of all segment in the image)}}{\text {number of SAM segments}} \end{aligned}$$

### Random Forest Classifier parameters

For training a Random Forest Classifier, we utilized a specific set of parameters to optimize performance for our dataset. The classifier comprised 100 trees (n_estimators=100), balancing performance and computational efficiency. We set the maximum depth of each tree (max_depth) to 10 to model complex patterns while avoiding overfitting. The minimum number of samples required to split an internal node (min_samples_split) was configured to 2. Similarly, the minimum number of samples required at a leaf node (min_samples_leaf) was set to 1.

We enabled bootstrap sampling (bootstrap = True) to construct trees, which enhances the diversity of the dataset each tree sees during training, improving generalization. The criterion for measuring the quality of splits was set to “gini” (criterion =“Gini”), a common choice for classification tasks. The number of features considered for the best split (max_features) was set to ’auto’, allowing the model to determine the optimal number of features automatically.

To ensure the reproducibility of results, we set a fixed random state (random_state = 42). Although our dataset was balanced, we chose a ’balanced’ approach for the class weight (class_weight = ’balanced’) to adjust weights inversely proportional to class frequencies automatically. Lastly, we opted not to use out-of-bag samples to estimate generalization accuracy (oob_score=False), focusing solely on in-sample accuracy metrics for model evaluation.

### Random Forest Classifier training pipeline

**Figure 7:**
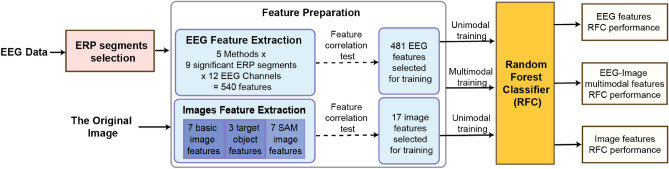
Random Forest Classifier training pipeline. This diagram illustrates the training of the Random Forest Classifier with three different sets of features: EEG, image, and a multimodal fusion of image and EEG features. The performance analysis of the classifier with each feature set is detailed in the Results section of our study.

The training pipeline, illustrated in Fig. 7, initiates with the processing of EEG data to select significant Event-Related Potential (ERP) components. Simultaneously, image features are extracted, including traditional Image-Based (IB) features and innovative features derived from the SAM, referred to as SAM features. The subsequent phase entails the preparation of features, where 540 unique EEG features are extracted from twelve channels across nine ERP segments using five methods, and 17 image features are extracted using three groups of methods. Feature selection for EEG and image features is then performed through a feature correlation test. This analysis selects 481 EEG and 17 image features for model training. In these selected EEG features, there is no pair of features with an absolute correlation of more than 0.8.

A Random Forest Classifier is trained using three distinct approaches for the classification task. In unimodal training, the classifier is individually trained with EEG features and separately with image features, and the respective performances being evaluated. Multimodal training combines the most significant EEG and image features to train the Random Forest Classifier to enhance prediction accuracy. The classifier’s performance is then gauged for each feature set to determine the effectiveness of unimodal versus multimodal feature applications in predicting outcomes.

The training was conducted on the UTS Interactive High-Performance Computing (iHPC) facility, equipped with an Nvidia Quadro GV100 GPU, 5120 CUDA cores, and 32 GB of HBM2 memory. 80% of the dataset was used for training and 20% for testing with 5-fold random stratified cross-validation applied. We reported the average classification result as the final result. The Hyperopt library version 0.2.7^55^ was used to optimise the hyperparameters, and the MLflow package version 2.3.2^56^ was used to save and compare the performance of the various models.

### Supplementary Information

Supplementary Information.

## Data Availability

The datasets used during this study are available from the corresponding author upon reasonable request.
