# Supplementary material for: Multimodal fusion for anticipating human decision performance
Source: Sci Rep. 2024 Jun 8;14:13217. doi: 10.1038/s41598-024-63651-2 (PMC11162455; doi:10.1038/s41598-024-63651-2)
Supplement: Supplementary file 1 — Supplementary Information. [file 41598_2024_63651_MOESM1_ESM.pdf]

Supplement Information

We employed the Independent Component Analysis (ICA) method to remove eye blinks in our analyses. However, we analyzed EEG data spanning 1000 ms, during which minor eye saccades—unremovable by ICA and not classified as noise—could confound the data. To determine if our model's performance is affected by information from eye saccades, we trained the Random Forest Classifier (RFC) using data from the F7 and F8 frontal channels, which are most susceptible to these artifacts. Initially, employing a methodology consistent with that described in the manuscript, we conducted a permutation statistics test with Bonferroni correction. This test revealed no significant difference in ERP components between Correct and Incorrect conditions, as illustrated in Figure 1. Consequently, we trained the RFC on the entire 1000 ms EEG segment from the F7 and F8 channels. We applied the same five feature extraction methods outlined in Table 4 of the paper. The performance of the RFC, as shown in Table 1, reveals that only subject S04's model accuracy exceeded the reference accuracy, with a mean accuracy of 0.72 ± 0.03. This outcome suggests that the model's performance is likely not influenced by the eye components in the data.

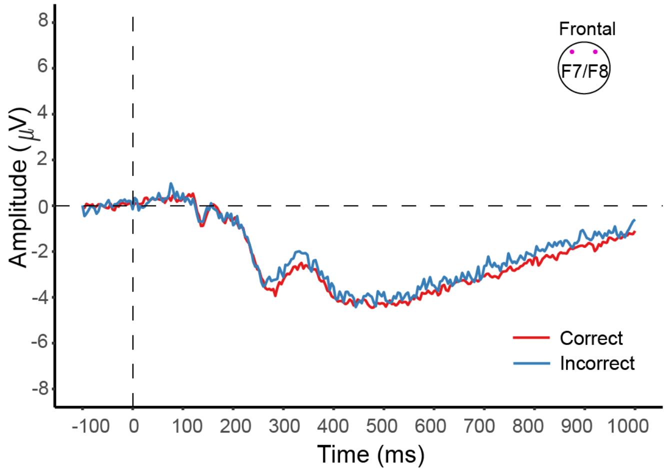

Figure 1. The ERP analysis for correct and incorrect response conditions in frontal F7 and F8 channels.

Table 1. Performance of the Random Forest Classifier trained in each dataset (subject level) using channels F7 and F8. These channels' 1000ms post-event EEG segment is applied to the EEG features extraction method and then trained with a Random Forest Classifier. Cells shaded in grey indicate subject datasets where the classifier did not surpass the reference accuracy (Ref Acc) with the respective feature sets.

Sub.	Acc	Pre	F1	Ref Acc
S01	0.71	0.72	0.74	0.84
S02	0.78	0.79	0.80	0.89
S03	0.70	0.70	0.72	0.81
S04	0.71	0.71	0.72	0.70
S05	0.72	0.73	0.73	0.83
S06	0.71	0.72	0.72	0.78
S07	0.68	0.70	0.70	0.75
S08	0.78	0.79	0.79	0.89
S09	0.74	0.74	0.75	0.87
S10	0.70	0.71	0.71	0.81
S11	0.69	0.69	0.70	0.81
S12	0.71	0.71	0.72	0.71
S13	0.72	0.72	0.73	0.76
S14	0.70	0.71	0.71	0.75
**Mean**	**0.72**	**0.73**	**0.73**	**0.80**
